# Genome-wide identification and characterization analysis of *CONSTANS-like* gene family in wheat (*Triticum aestivum* L.)

**DOI:** 10.3389/fpls.2025.1646979

**Published:** 2025-10-16

**Authors:** Yameng Gao, Ziqi Wang, Wenjie Kan, Zhu Yang, Zhiwei Li, Shuangling Jian, Dacheng Wang, Caiguo Tang, Lifang Wu

**Affiliations:** ^1^ The Center for Ion Beam Bioengineering & Green Agriculture, Hefei Institutes of Physical Science, Chinese Academy of Sciences, Hefei, Anhui, China; ^2^ College of Basic Medical Sciences, Anhui Medical University, Hefei, China; ^3^ Science Island Branch, University of Science and Technology of China, Hefei, Anhui, China; ^4^ College of Life Sciences, Anhui Agricultural University, Hefei, Anhui, China

**Keywords:** wheat, CONSTANS-Like, stress, co-expression network, subcellular localization

## Abstract

The CONSTANS-like (COL) proteins are plant-specific transcription factors that play pivotal roles in growth, development, stress responses, and photoperiodic flowering. However, the *CONSTANS-like* (*TaCOL*) gene family in wheat (*Triticum aestivum*) remains inadequately characterized. In this study, we systematically identified 51 *TaCOL* genes in the wheat genome and classified them into three phylogenetic subfamilies (I, II, and III). Members within each subfamily shared conserved gene structures and motif compositions. Chromosomal location analysis revealed that the *TaCOL* genes were distributed across 15 chromosomes, with segmental duplication events identified as a major driver of this family expansion. Collinearity analysis among eight other Poaceae species further suggested that the *TaCOL* gene family was highly conserved and had undergone strong purifying selection during evolution. Promoter analysis uncovered numerous light-responsive and stress-related cis-elements, suggesting roles in environmental adaptation. Expression profiling demonstrated both tissue-specific and developmental stage-dependent patterns, and co-expression network analysis linked certain *TaCOL* genes to stress response and floral development pathways. Using qRT-PCR, we examined the expression of *TaCOL* genes under long-day and short-day photoperiods, revealing distinct expression patterns of several genes, including *Ta-2B-COL4*, *Ta-5D-COL16*, and *Ta-7D-COL48*. Furthermore, subcellular localization and transcriptional activation assays confirmed that the three proteins were nuclear localized and that Ta-5D-COL16 exhibited transcriptional activation activity. Together, these results provided valuable insights into the evolutionary history and molecular functions of *TaCOL* genes, establishing a foundation for future functional studies aimed at elucidating their roles flowering time regulation and environmental adaptation in wheat.

## 1 Introduction

Throughout the evolutionary history, plants have developed sophisticated regulatory systems to coordinate growth, development, and responses to environmental conditions. A crucial process within this framework is flowering, which is meticulously controlled by multiple interconnecting pathways. These include the photoperiod, gibberellin, vernalization, autonomous, environmental temperature, and age pathway (Boss et al., 2004; Amasino and Michaels, 2010; Fornara et al., 2010; Srikanth and Schmid, 2011). In *Arabidopsis thaliana* (*A. thaliana*), the *CONSTANS* (*CO*) gene, as the member in Group I of *CONSTANS-like* gene family, is a pivotal component of the photoperiod pathway. It serves to convert light and circadian clock signals into flowering signals. Through this conversion, *CO* orchestrates the transcriptional activation of genes such as *FLOWERING LOCUS T* (*FT*) and *SUPPRESSOR OF OVEREXPRESSION OF COL 1* (*SOC1*), playing a decisive role in the transition to flowering and the formation of inflorescences (Putterill et al., 1995; Graeff et al., 2016; Schmid et al., 2003).

The CONSTANS-like protein generally comprises one or two conserved B-box domains (B-box 1 and B-box 2) at the N-terminus. These domains, characterized by a specific cysteine residue pattern (C-X2-C-X16-C-X2-C), are crucial for protein interactions, especially in light signal responses. The C-terminus features a CCT domain (named after CO, CO-like, and TOC1), approximately 43 amino acids in length, which is associated with nuclear localization and DNA binding (Chaurasia et al., 2016). The functions of *CONSTANS-like* genes have been identified and elucidated in model plants. For instance, the *CONSTANS*-like family comprises17 members in *A. thaliana*, 16 in *Oryza sativa*, 13 in *Beta vulgaris*, 9 in *Hordeum vulgare*, and 4 in *B. napus* (Robson et al., 2001; Griffiths et al., 2003; Chia et al., 2008; Robert et al., 1998). Specifically, *AtCOL4* (Steinbach, 2019), *AtCOL8* (Takase et al., 2011), and *AtCOL9* (Cheng and Wang, 2005) act as floral repressors under long days (LD), whereas *AtCOL5*, akin to *CO*, accelerates flowering (Hassidim et al., 2009). In rice, the CO ortholog *Hd1* (Heading date 1) promotes flowering by inducing *Hd3a* under short days (SD), yet suppresses it via interaction with Grain number, plant height, and heading date 7 (Ghd7) and Days to Heading 8 (DTH8) under LD. Transgenic lines over-expressing *OsCO3* flower late under SD owing to repressed FT-like genes, while the *dhd4* mutant heads slightly earlier than the wild type under natural LD without yield penalty (Cai et al., 2021). *OsCOL10* and *OsCOL16* also negatively regulate *FT* paralogues through the *Ghd7*-*Ehd1* module under both SD and LD (Tan et al., 2016; Wu et al., 2017).

The *CO* ortholog also plays a key role in wheat and barley photoperiod pathways. Under LD, *Ppd-H1* is first activated and immediately up-regulates *CO*; *CO* then directly induces *VERNALIZATION 3* (*VRN3*). Before vernalization, *VRN2* represses *VRN3*, but *CO* activity overrides this brake, allowing *VRN3* to activate *VRN1* and trigger flowering (Li and Xu, 2017). In barley, the *CO* paralogues *HvCO1* and *HvCO2* promote *FT-like* expression in an LD-dependent manner. The B-box 2 mutation in *HvCO1* delays flowering under short days (SD), underscoring the functional necessity of the B-box domain (Tamaki et al., 2007). Consistently, *HvCO1* over-expression accelerates heading in both LD and SD. By contrast, in tetraploid wheat the *CO1* and *CO2* copies act as weak repressors of heading under either photoperiod. Nevertheless, *CO1* can fine-tune flowering in *PPD1*-deficient backgrounds, revealing a dosage-sensitive modulatory role (Shaw et al., 2020).

Beyond the canonical role in flowering time, *CONSTANS-like* genes have been co-opted for diverse developmental and stress-adaptive processes. In the long-day plant *Arabidopsis* and the short-day plant soybean, *CO* controls seed size by modulating seed-coat epidermal cell proliferation through the photoperiod-dependent *CO-AP2* pathway (Yu et al., 2023). AtCOL3 promotes photomorphogenesis, interacts with BBX32 to fine-tune light signaling and flowering, and regulates lateral branching and root growth independently of COP1 (Datta et al., 2006; Tripathi et al., 2017). Under high R:FR light, *TaCOL7* enhances wheat tillering by reducing auxin levels and activating *SUR2* expression, whereas *AtCOL7* mediates *phyB*-dependent shade avoidance (Zhang et al., 2014). In tomato, SlCOL1 stabilizes GLK2 to control chlorophyll accumulation in immature fruit (Yang et al., 2020). OsCOL9 interacts with OsRACK1 to enhance salicylic acid and ethylene signaling pathways, which improves rice blast resistance (caused by *Magnaporthe oryzae*) (Liu et al., 2016a). In wheat, the dominant allele *TaCOL-B5* increases grain yield by up to 19.8% (Zhang et al., 2022b), and three COL-zinc-finger loci (*TraesCS7D02G209000*, *TraesCS7D02G213000* and *TraesCS7D02G220300*) lie within QTL intervals for heading date and plant height (Xu et al., 2022). Stress-tolerance functions are equally prevalent: *GmCOL1a* alleviates salt and drought stress in soybean, while heterologous expression of mango *MiCOL2A/B* or apple *MdCOL9* enhances *Arabidopsis* abiotic-stress tolerance (Liang et al., 2023; Chen et al., 2022). Conversely, *BnCOL2* from *Brassica napus* and rice *Ghd2* reduce drought tolerance when over-expressed in *Arabidopsis* (Liu et al., 2020a, 2016b). Collectively, these functions underscore the *COL* gene family as multifaceted regulators that integrate environmental cues to optimize plant growth, development and stress resilience.

*CONSTANS-like* (COL) genes have been extensively characterized in model eudicots and monocots, with curated inventories of 17 in *A. thaliana* (Griffiths et al., 2003), 16 in rice (Griffiths et al., 2003), 19 in maize (Song et al., 2018), 11 in *Setaria italica* (Jiang et al., 2024), 20 in cucumber (Tian et al., 2021), 20 in radish (Hu et al., 2018), 13 in tomato (Yang et al., 2020) and 15 in potato (Li et al., 2023). However, the *COL* family in bread wheat remains poorly characterized. As a hexaploid species (AABBDD, 16 Gb) that feeds more than one-third of the global population, wheat presents both a formidable genomic challenge and an unparalleled opportunity to dissect gene-family evolution and functional redundancy. Nevertheless, a systematic analysis of the complete *TaCOL* gene complement is still lacking. Given the complexity of the wheat genome and the potential for functional redundancy among *TaCOL* members, a genome-wide comprehensive study is essential to elucidate the roles of this gene family in wheat growth, development, and stress responses.

In this study, we conducted a comprehensive and systematic analysis of the *TaCOL* gene family in wheat, including the identification of all *TaCOL* genes, along with detailed analyses of their phylogenetic relationships, gene structures, motif compositions, chromosomal distributions, and duplication events. We also conducted transcriptome profiling across various tissues, developmental stages, and under abiotic stress conditions. Furthermore, co-expression networks were constructed to uncover potential target genes regulated by *TaCOL* genes, many of which were implicated in flower development. However, the functional roles of these interactions require further experimental validation. The significance of this study lies in its potential to offer novel insights into the evolutionary dynamics and functional diversification of the CONSTANS-like gene family in wheat. These findings will help elucidate how wheat adapts to varying environments and optimizes flowering time, thereby providing valuable information for breeding programs aimed at developing varieties with enhanced adaptability to diverse growing conditions.

## 2 Materials and methods

### 2.1 Identification and information collection of CONSTANS-like genes in wheat

The coding sequence (CDS), protein sequence, genome sequences, and gene sets of *T. aestivum* were retrieved from Ensembl Plants website (http://plants.ensembl.org/info/data/ftp/index.html). Subsequently, a total of 79 CONSTANS-like amino acid sequences, comprising 17 from *A. thaliana*, 16 from *O. sativa*, 18 from *Z. mays*, 14 from *Sorghum bicolor*, and 14 from *Phyllostachys edulis*, were employed as queries to perform a local BLASTP search (E-value < 1e-5) in the *T. aestivum* protein database (Griffiths et al., 2003). The amino acid sequences corresponding to the resulting identity names were subsequently submitted to the NCBI Conserved Domain Database (https://www.ncbi.nlm.nih.gov/Structure/cdd/wrpsb.cgi) to verify the presence of both CCT and B-box domains (Huang et al., 2021). Following chromosomal order, each *CONSTANS-like* gene was assigned a unique name. Sequence statistics were computed with the Fasta Stats function of TBtools, and the molecular weight and theoretical isoelectric point of all CONSTANS-like proteins were batch-calculated via ExPASy (https://web.expasy.org/compute_pi/) (Wilkins et al., 1999). Subcellular localizations were predicted using Plant-mPLoc (http://www.csbio.sjtu.edu.cn/bioinf/plant-multi/) (Chou and Shen, 2010).

### 2.2 Phylogenetic tree, motif composition, and gene structure

Multiple sequence alignment of CONSTANS-like proteins was performed with ClustalW under default parameters (Thompson et al., 1994), and a neighbor-joining phylogenetic tree was constructed in MEGA11 with 1,000 bootstrap replicates (Tamura et al., 2021). Gene structures were visualized by uploading coding and genomic sequences to GSDS 2.0 (http://gsds.gao-lab.org/) (Hu et al., 2015). Conserved motifs were identified using MEME (Bailey et al., 2015) with the following settings: motif width 6–100 aa, maximum number of motifs = 10. HMMER was employed for additional domain verification (Potter et al., 2018).

### 2.3 Chromosomal distribution and microcollinearity analysis

Synteny analysis was performed with MCScanX (TBtools) using the wheat genome and GFF file (BLASTP E-value ≤ 1e-5, max five hits per locus) (Wang et al., 2012), and the resulting blocks were visualized with Advanced Circos. Genome assemblies and annotations for *Triticum dicoccoides*, *Aegilops tauschii*, *H. vulgare*, *Secale cale*, and *S. italica* were downloaded from Ensembl Plants (http://plants.ensembl.org); those for *S. bicolor* and *O. sativa* were obtained from Phytozome (https://phytozome-next.jgi.doe.gov/), and the *Z. mays* was retrieved from MaizeGDB (https://www.maizegdb.org/). Collinearity plots were generated with the Multiple Synteny Plot tool in TBtools (Chen et al., 2020). Non-synonymous (Ka) and synonymous (Ka) substitution rates of duplicated gene pairs were calculated with the Simple Ka/Ks Calculator (NG) implemented in TBtools (Chen et al., 2020; Gao et al., 2021a) and visualized in GraphPad Prism 8.3.0. Ka/Ks > 1, = 1 and < 1 indicate positive, neutral and purifying selection, respectively. The species timetree was constructed using divergence time data from TimeTree (http://timetree.org/) (Kan et al., 2025).

### 2.4 Cis-acting element analysis

The 2000bp sequence upstream of the CDS of *TaCOLs* was extracted from the wheat genome and defined as the corresponding gene promoter. Subsequently, the promoter sequences were submitted to the PlantCARE website (http://bioinformatics.psb.ugent.be/webtools/plantcare/html/) to identify various cis-acting regulatory elements (Lescot et al., 2001). These elements were classified into three main categories: plant growth and development, phytohormone response, and biotic and abiotic stress response (Gao et al., 2018).

### 2.5 Expression profile

To obtain and analyze wheat gene expression profiles under diverse conditions, multiple datasets were accessed and integrated. Gene expression data across five tissues (root, stem, leaf, spike, and grain), four developmental stages (booting, heading, anthesis, and grain-filling), two temperature conditions (4°C and 23°C) were retrieved from the WheatOmics platform (http://wheatomics.sdau.edu.cn/) (Ma et al., 2021). Additionally, RNA-seq data under various abiotic stresses, including heat (H), drought (D), combined heat and drought (HD), salt (S), salt-drought (SD), salt-heat (SH), and salt-heat-drought (SHD) stresses, were downloaded from the GEO dataset GSE183007 and aligned to the Chinese Spring wheat reference genome (Da Ros et al., 2023). Data for polyethylene glycol (PEG) and abscisic acid (ABA) treatments were also acquired from the NCBI Sequence Read Archive (SRA). All raw sequencing reads were uniformly pre-processed and aligned to the Chinese Spring wheat genome with TBtools (Chen et al., 2020). Expression values were normalized and subjected to hierarchical clustering analysis, with results visualized as heatmaps. The corresponding SRA accession numbers were provided in **Supplementary Table S5**.

### 2.6 Co-expression network construction and gene annotation

A co-expression network was constructed using the WGCNA R package on the RStudio platform based on transcriptome data from 32 wheat samples subjected to abiotic stress conditions (Langfelder and Horvath, 2008; Yu et al., 2020). The dataset comprised eight stress treatment groups, each with four biological replicates. The resulting network was visualized using Cytoscape. Gene Ontology (GO) enrichment analysis was performed utilizing the Triticeae-Gene Tribe database (http://wheat.cau.edu.cn/TGT/), with results presented as bubble charts; detailed characteristics of each co-expression module were summarized in **Supplementary Table S7**. Additionally, Kyoto Encyclopedia of Genes and Genomes (KEGG) pathway analysis was carried out via eggNOG-mapper (http://eggnogmapper.embl.de/) for functional annotation of protein sequences, and the outcomes were visualized using bar charts.

### 2.7 Plant treatment and quantitative real-time PCR (qRT-PCR) analysis

*T. aestivum* cv.Chinese Spring wheat seeds were surface-sterilized with 5% sodium hypochlorite and sown in nutrient soil in a greenhouse. The greenhouse conditions were set at 23 ± 1°C, with a 12 h light/12 h dark photoperiod, 60-70% relative humidity, and 6000 lx light intensity. After two weeks, seedlings were transferred to two different photoperiod regimes: 16 h light/8 h dark and 8 h light/16 h dark (Zhao et al., 2022; Huang et al., 2022; Yang et al., 2020). Leaves were collected at 4-hour intervals over a 24-hour period under each photoperiodic condition, immediately frozen in liquid nitrogen and stored at -80°C.

Total RNA was extracted using the RNA Rapid Extraction Kit (Mei5bio, MF610-01). First-strand cDNA synthesis was conducted with the MonscriptTM RTIII All-in-One Mix with dsDNase. Primers were designed using Primer Premier 5.0 software and validated via NCBI-Primer-BLAST. TaGAPDH (GI: 7579063) served as the internal reference gene (Tang et al., 2020). qRT-PCR was performed using the 2× Quantinova SYBR Green PCR Master Mix under the following conditions: 95°C for 5 min, 45 cycles of 72°C for 20 s, and a melting curve. Relative gene expression was calculated using the 2^–ΔΔCt^ method and presented using GraphPad Prism 8.3.0 (Lei et al., 2021). One-way analysis of variance (ANOVA) was used via Duncan’s test at p < 0.05 in IBM SPSS v25.0 to compare significant differences in different time point within each treatment (Gao et al., 2021b). The relevant genes and their primer sequences were listed in **Supplementary Table S8**.

### 2.8 Subcellular localization and self-activating detection in yeast

The CDS of *TaCOL* genes, excluding stop codons, were amplified from a cDNA library derived from Chinese Spring wheat and subsequently cloned into the Simple vector (TransGen, Beijing, China) for sequencing. The PCR products were fused with linearized vectors via homologous arms. We digested the pCAMBIAI1305 and pGBKT7 vectors with the restriction enzyme pairs *Xba*I/*BamH*I and *EcoR*I/*BamH*I, respectively. All primers used in this study are listed in **Supplementary Table S9**.

The pCAMBIA1305 vector contains a 35S-driven GFP sequence. The *TaCOL*-GFP and control vectors were introduced into *Agrobacterium tumefaciens* GV3101 (Weidi, Shanghai, China). These *Agrobacterium* strains were used to infiltrate *Nicotiana benthamiana* leaves. After 40 hours, leaves were stained with DAPI and observed under an Olympus SpinSR10 microscope (Japan) (Kan et al., 2025). The pGBKT7 vector, which expresses proteins as fusions to the GAL4 DNA-binding domain (BD), was used in this study. The recombinant plasmid pGBKT7-TaCOL, along with negative control (empty pGBKT7 vector) and positive control (pGBKT7-53 + pGADT7-T), was transformed into Y2HGold competent yeast cells (Weidi, Shanghai, China) using the PEG/LiAc method. Following transformation, yeast cells were selected on synthetic dropout (SD) medium lacking tryptophan (SD/-Trp) to confirm bait plasmid retention. To assess possible autoactivation of the bait protein, transformed yeast were also plated on a higher stringency medium lacking tryptophan, histidine, and adenine, and supplemented with X-α-Gal (SD/-Trp/-His/-Ade/X-α-Gal). Growth and blue coloration on this medium would indicate transcriptional activation activity of the MEL1 reporter via α-galactosidase (Kan et al., 2025).

## 3 Results

### 3.1 Identification and physicochemical characterization of *CONSTANS-like* genes in wheat

**Figure 1:**
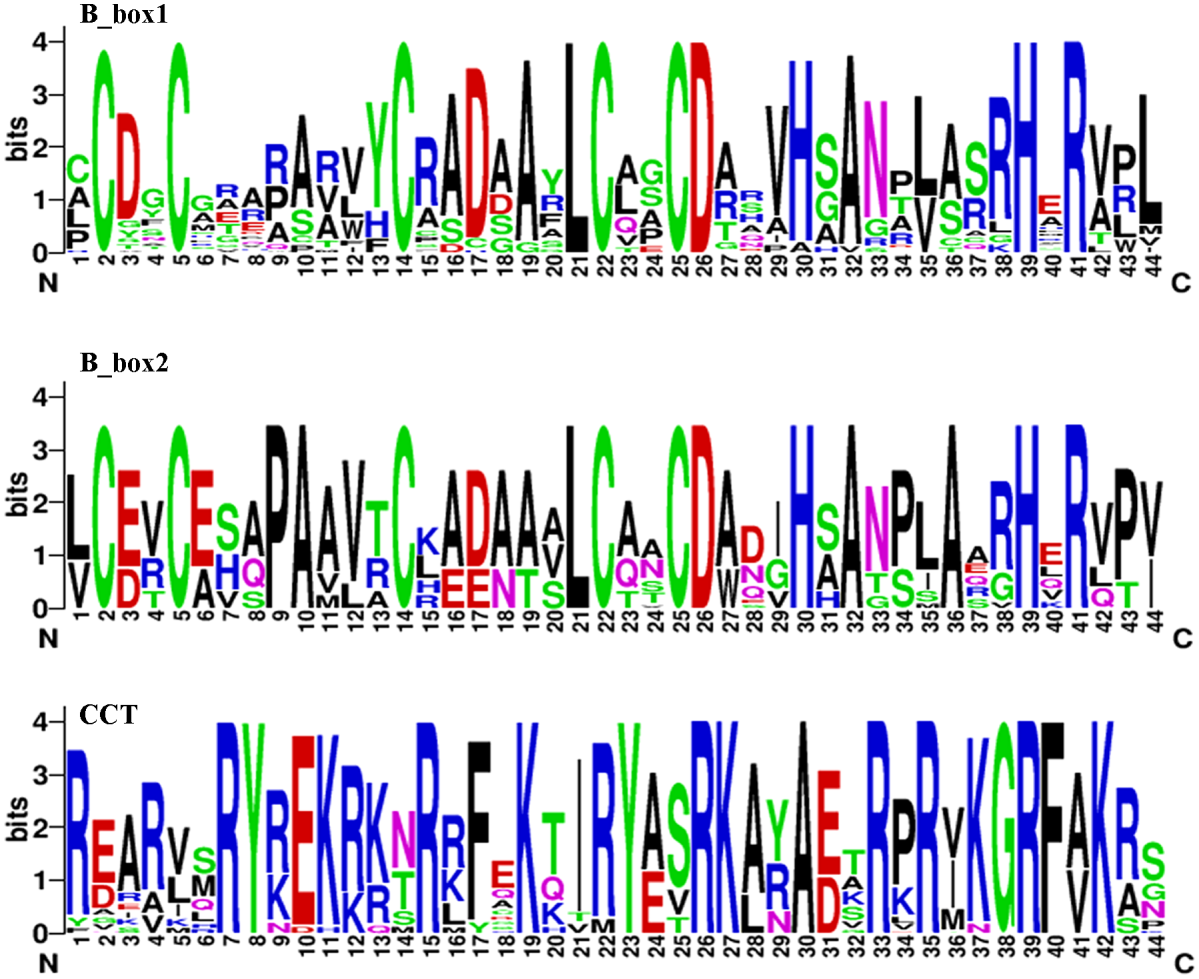
Sequence logos for the conserved domains of B-box1, B-box2, and CCT. The B-box1 domain had the sequence C-C-C-L-C-C-D-H-A-H-R. B-box2 was characterized by the sequence C-C-P-A-C-L-C-C-D-H-A-A-H-R. The CCT domain was rich in arginine residues, with the sequence R-RY-EK-R-F-K-RY-RK-A-R-R-KGRF-K.

Fifty-one candidate members were identified in the *T. aestivum* protein database via BLASTP. It was confirmed that they all contained at least one B-box domain and a complete CCT domain. Seventeen of these proteins featured two B-box domains: the B-box1 domain had the sequence C-C-C-L-C-C-D-H-A-H-R, while B-box2 was C-C-P-A-C-L-C-C-D-H-A-A-H-R. The CCT domain, rich in arginine (R): R-RY-EK-R-F-K-RY-RK-A-R-R-KGRF-K (**Figure 1**), was also conserved and aligns with prior studies (Robson et al., 2001). The largest protein was Ta-7A-COL37, with 490 amino acids. It also had the longest CDS (1473 bp) and the highest molecular weight (52005.04 Da). The theoretical isoelectric points of the 51 proteins ranged from 4.79 (Ta-6D-COL30) to 7.65 (Ta-7A-COL33). And all *TaCOL* proteins were predicted to be nuclear-localized. The specific and detailed information was shown in **Supplementary Table S1**.

### 3.2 Phylogenetic relationship, gene structure, and motif compositions

**Figure 2:**
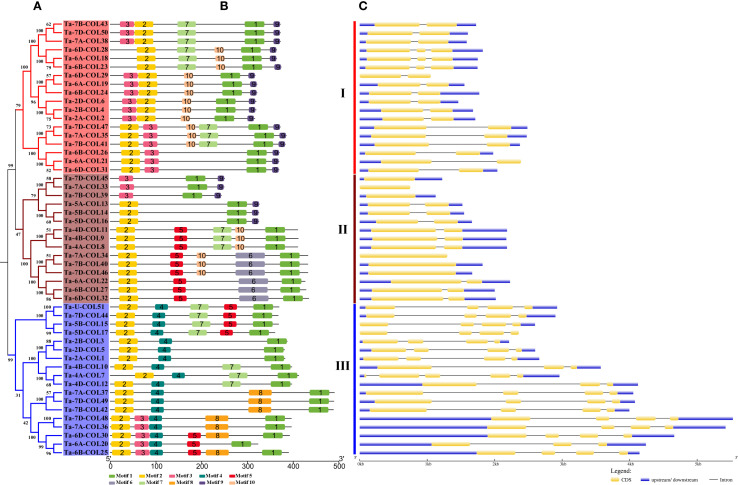
Phylogenetic relationships, conserved motifs and gene structure of 51 TaCOLs. **(A)** Phylogenetic analysis of 51 TaCOL proteins was performed using MEGA11 software. **(B)** Motif analysis of TaCOL proteins revealed 10 conserved motifs, numbered 1–10 and represented by different colored boxes. **(C)** The exon/intron structure of *TaCOLs* was analyzed. Yellow boxes denoted exons, gray lines indicated introns, and blue boxes represented the untranslated 5′- and 3′-regions.

To classify these *TaCOLs* more accurately, a comprehensive phylogenetic tree was constructed using 116 protein sequences from *A. thaliana* (17), *O. sativa* (16), *Z. mays* (18), *P. edulis* (14), and *T. aestivum* (51). Consistent with Hu et al. (2018), these CONSTANS-like members were divided into three distinct subfamilies (**Supplementary Figure S1**). Meanwhile, the 51 full-length TaCOL proteins were also classified into three subfamilies (I, II, and III) based on the phylogenetic tree (**Figure 2A**). Subfamily I and subfamily II each comprised 18 members, and subfamily III consisted of 15 members. Motif 1 corresponded to the CCT domain; motif 2 and motif 3 were linked to B-box domains (**Supplementary Table S2**). This explained why all members contained motif 1, along with either motif 2 or motif 3, or all three motifs collectively. In subfamily I, three TaCOL proteins (Ta-6D-COL28, Ta-6A-COL18, Ta-6B-COL23) harbored motif 2 but lacked motif 3, whereas the remaining 15 members possessed both motifs in a variable arrangement (**Figure 2B**). Motif 9 occurred in this subfamily and six members of subfamily III, invariably residing at the C-terminus. In subfamily II, five TaCOL proteins retained both motif 2 and motif 3, whereas the remaining 13 members possessed only motif 2, signifying a single B-box domain. Motif 4 was ubiquitous throughout the subfamily, whereas motif 8 was restricted to a subset of members; these diagnostic motifs likely underlay the delineation of distinct subfamilies (**Figure 2A, B**). In terms of genetic structure, the number of exons among the 51 *TaCOL* genes ranged from one to four. Notably, six *TaCOLs* within subfamily III were intronless, while the remaining nine members each contained one intron. Members of subfamily I typically contained one to two introns, whereas those in subfamily II generally harbored three introns, with the exceptions of *Ta-4A-COL7* and *Ta-4D-COL12*. Overall, each subfamily exhibited a largely conserved motif compositions and gene architecture, alongside subtle variations.

### 3.3 Chromosome mapping and collinearity analysis of *TaCOL* genes

**Figure 3:**
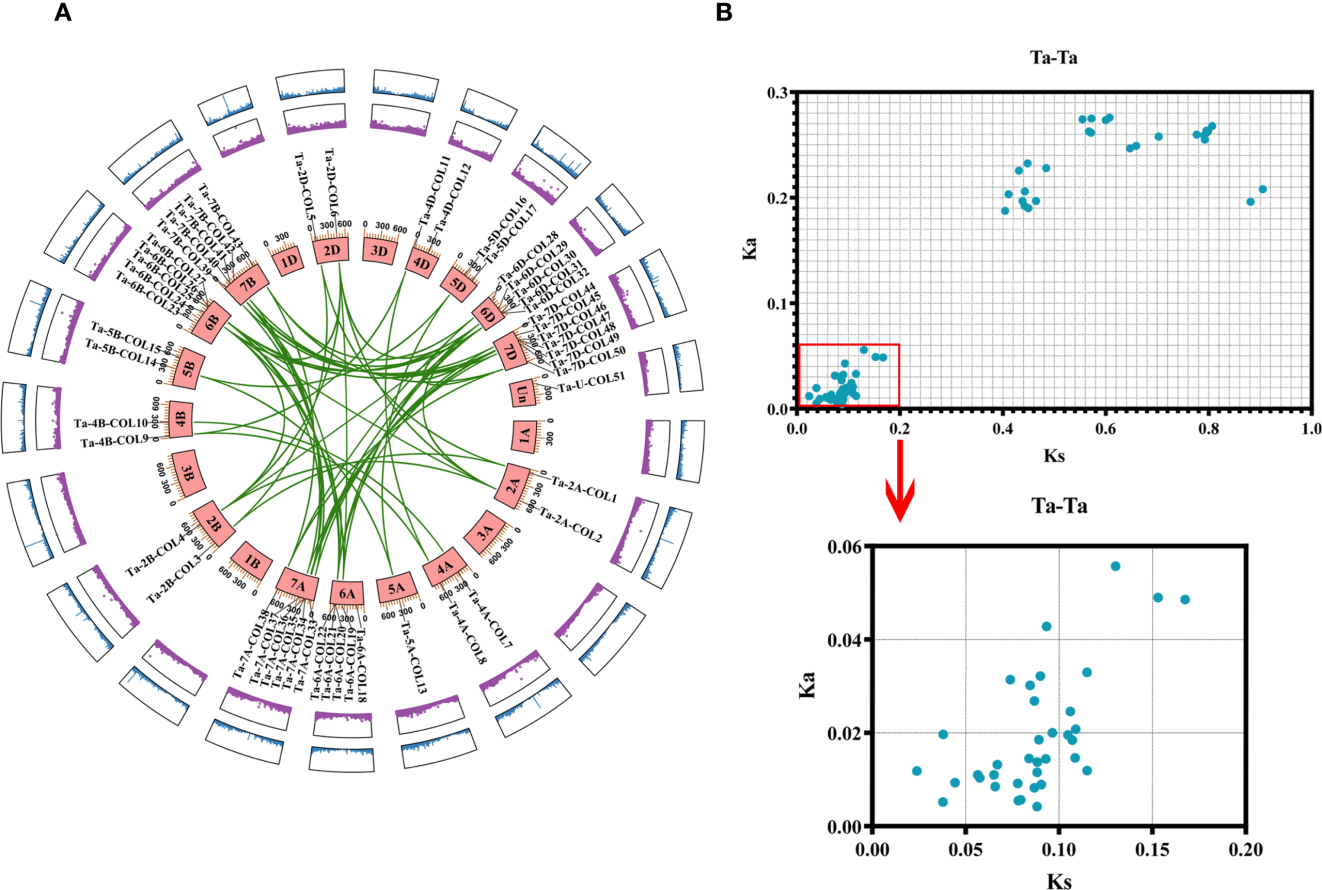
Chromosome mapping and gene duplication analysis of *TaCOLs*. **(A)** The distribution of *TaCOLs* across 21 chromosomes was shown, with chromosome numbers labeled on the chromosome block. Paralogous pairs resulting from segmental duplications were connected by green lines. The outer purple and blue boxes represent the gene density on the chromosomes. **(B)** The scatterplot displayed the Ka and Ks values for 64 paralogous pairs (Ta-Ta).

The common hexaploid bread wheat (*Triticum aestivum*) possesses a heterohexaploid genome (AABBDD, 2n = 6x = 42) consisting of three distinct subgenomes A, B, and D; each subgenome derived from different wild grass progenitors. The A subgenome originated from the diploid wheat *Triticum urartu* (AA). Tetraploid wheat (*Triticum turgidum*, AABB) subsequently arose from a hybridization event between *T. urartu* (AA) and *Aegilops* sp*eltoides* (BB). Finally, hexaploid bread wheat was formed through a second hybridization between tetraploid wheat and the diploid wild relative *A. tausch*ii (DD) (null et al., 2014). Among the 51 *TaCOL* genes, the majority were mapped to 15 chromosomes, with a subset located at unknown chromosomal positions (Un). None of the *TaCOL* genes were present on chromosomes 1 and 3 of the A, B, and D subgenomes. The high number of *TaCOL* genes was observed on chromosomes 6A (5 *TaCOLs*), 6B (5 *TaCOLs*), 6D (5 *TaCOLs*), 7A (6 *TaCOLs*), 7B (5 *TaCOLs*), and 7D (7 *TaCOLs*) (**Figure 3A**). And we identified 64 pairwise duplications among the 51 *TaCOLs*, indicating that most genes exist as two or more homologs. Specifically, 17 *TaCOLs* were present as pairs; six (*Ta-6A-COL19*, *Ta-6D-COL28*, *Ta-7A-COL38*, *Ta-7B-COL43*, *Ta-7D-COL48* and *Ta-7D-COL50*) had three paralogous genes; seven (*Ta-2A-COL2*, *Ta-2B-COL4*, *Ta-6A-COL21*, *Ta-6B-COL26*, *Ta-6D-COL31*, *Ta-7A-COL35* and *Ta-7B-COL41*) possessed four homologous genes; and nine (*Ta-2D-COL6*, *Ta-6A-COL22*, *Ta-6B-COL24*, *Ta-6B-COL27*, *Ta-6D-COL29*, *Ta-6D-COL32*, *Ta-7A-COL34*, *Ta-7B-COL40* and *Ta-7D-COL46*) retained five homologous genes (**Supplementary Table S3**). The maximum Ka and Ks values for all homologous pairs were 0.275 and 0.904, respectively (**Figure 3B**; **Supplementary Table S3**). And the Ka/Ks value was significantly below 1, indicating that the *TaCOL* genes were under strong purifying selection.

**Figure 4:**
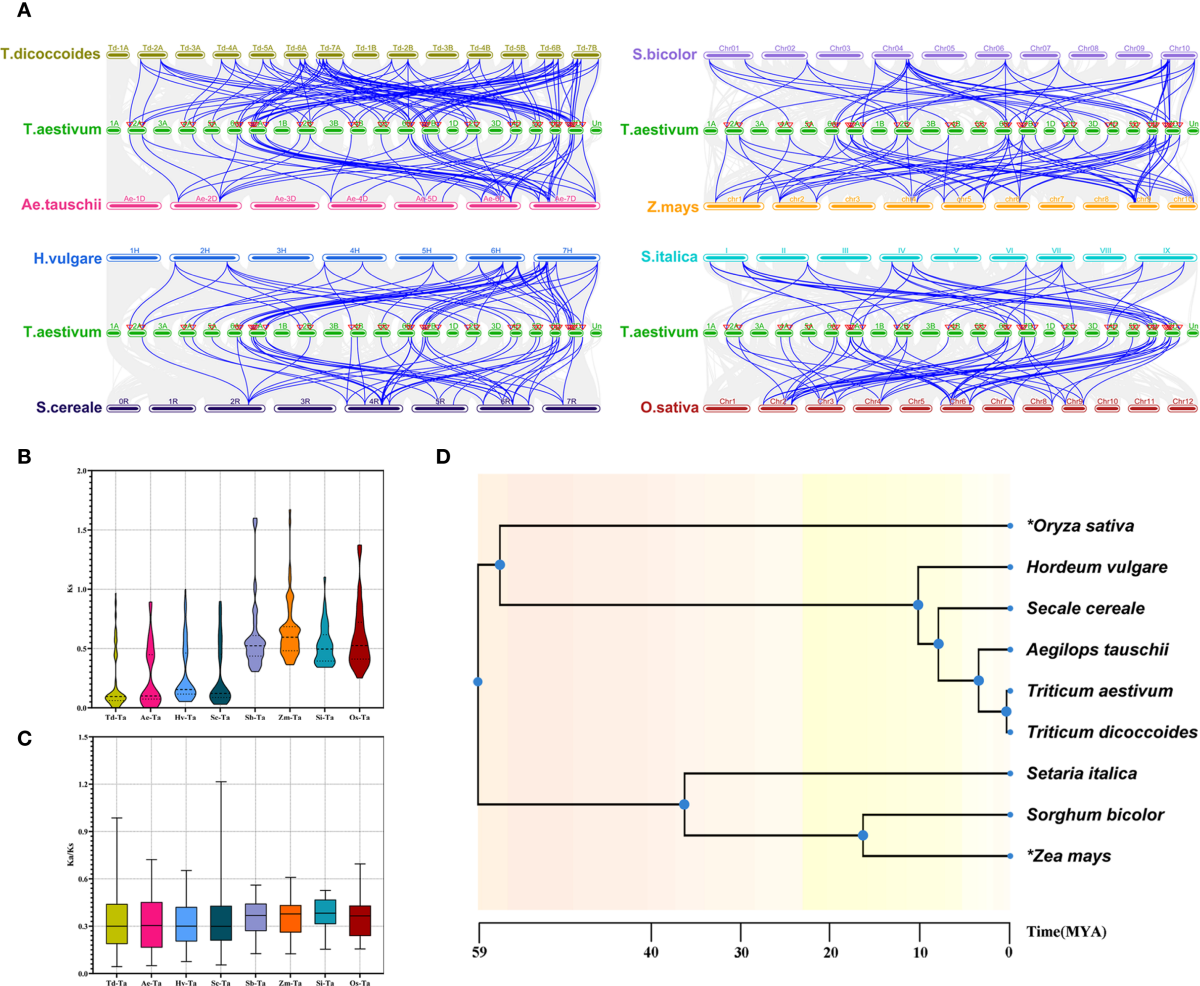
The synteny analysis of *TaCOLs* between *T. aestivum* and eight other plant species, including *T. dicoccoides*, *A. tauschii*, *H. vulgare*, *S. cereal*e, *S. italica*, *S. bicolor*, *O. sativa*, and *Z. mays*. **(A)** Gray lines in the background indicated collinear blocks within the *T. aestivum* and other plant genomes. Blue lines highlighted the syntenic *COL* gene pairs between *T. aestivum* and the other eight plant species. **(B, C)** Violin plots of Ks and Ka/Ks values for syntenic gene pairs between *T. aestivum* and the eight species. **(D)** Interspecies temporal evolutionary divergence tree between *T. aestivum* and the eight species.

To further explore the evolutionary relationships of *CONSTANS-like* genes, we conducted a collinearity analysis of *T. aestivum* and eight other Poaceae plants (*T. dicoccoides*, *A. tauschii*, *H. vulgare*, *Secale cereale*, *S. italica*, *S. bicolor*, *O. sativa*, and *Z. mays*) (**Figure 4A**). Despite considerable differences in the number of orthologous gene pairs, the number of associated *TaCOLs* remained relatively comparable. The orthologous pair distributions were as follows: Td-Ta (96 pairs involving 38 *TaCOLs*), Ae-Ta (46 pairs involving 33 *TaCOLs*), Hv-Ta (63 pairs involving 43 *TaCOLs*), Sc-Ta (58 pairs involving 40 *TaCOLs*), Sb-Ta (47 pairs involving 32 *TaCOLs*), Zm-Ta (71 pairs involving 34 *TaCOLs*), Si-Ta (40 pairs involving 32 *TaCOLs*), and Os-Ta (65 pairs involving 40 *TaCOLs*). And the average Ks values of these orthologous pairs were as follows: Td-Ta (0.203016013), Ae-Ta (0.26228061), Hv-Ta (0.281602287), Sc-Ta (0.293103976), Si-Ta (0.527560353), Os-Ta (0.595051996), Sb-Ta (0.602337798) and Zm-Ta (0.652450481) (**Figure 4B**; **Supplementary Table S3**). The Ka/Ks ratios were less than 1, except for a maximum value of 1.2 in *S. cereale* (**Figure 4C**; **Supplementary Table S3**). Additionally, the time trees of the nine Poaceae plants revealed that wheat diverged most recently from *T. dicoccoides*, followed by *A. tauschii*, *S. cereale*, *H. vulgare*, *O. sativa*, and finally *S. italica*, *S. bicolor*, and *Z. mays* (**Figure 4D**). This divergence order largely mirrored the trend of mean Ks values, the sole exception being the average Ks observed in rice.

### 3.4 Cis−acting elements analysis in promoter region of *TaCOL* genes

**Figure 5:**
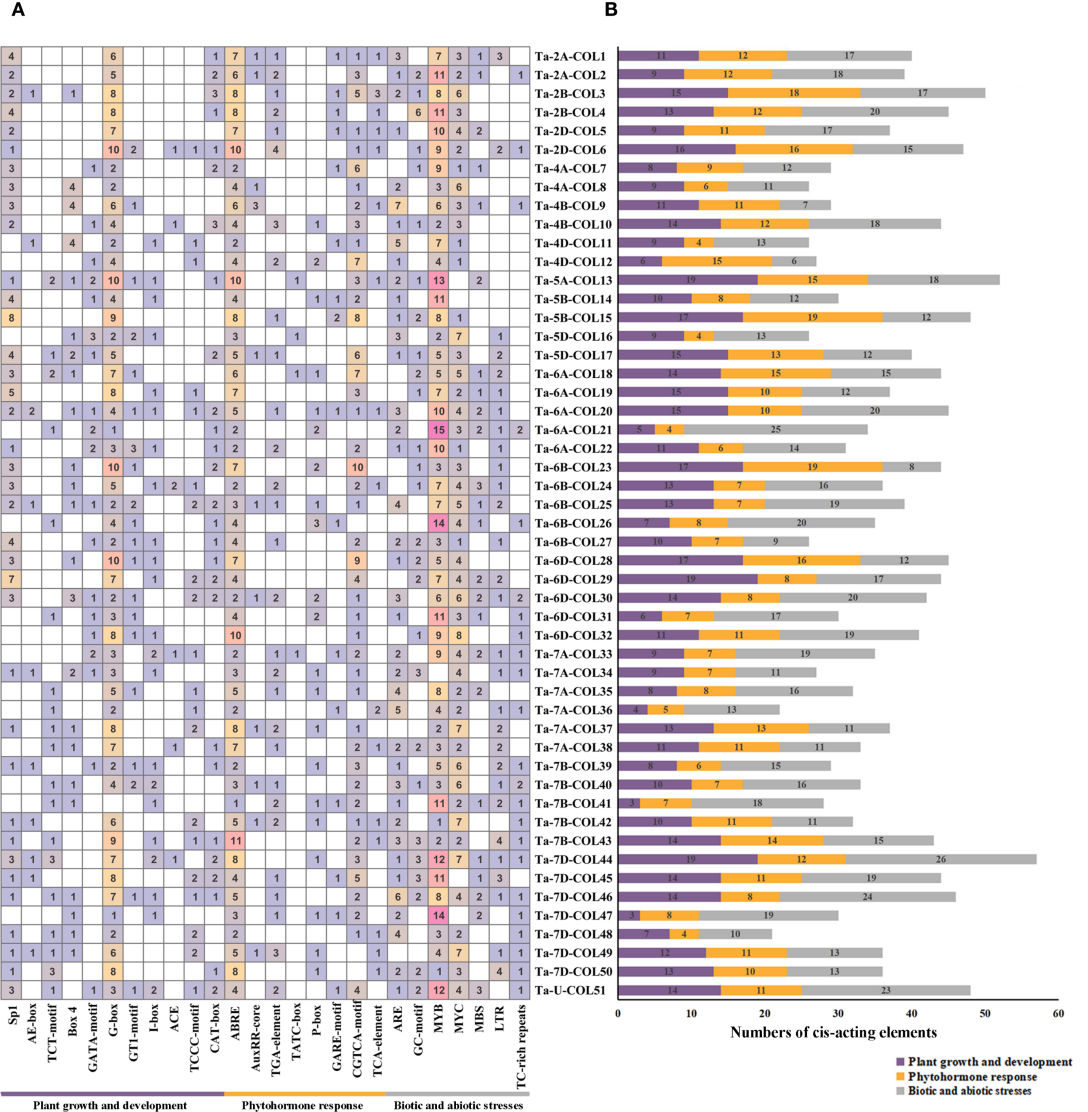
Analysis of cis-acting elements in *TaCOL* gene promoters. **(A)** The number of cis-acting elements in the promoter regions of *TaCOL* genes was shown. **(B)** The total number of three types of promoters was presented as a bar chart.

To elucidate the potential regulatory functions of the 51 *TaCOLs*, we investigated the cis-regulatory elements in their promoter regions and systematically classified them into three main categories: plant growth and development, phytohormone response, and biotic and abiotic stresses (**Figure 5**; **Supplementary Table S4**). Light-responsive cis-acting elements encompass a wide range of types, such as Sp1, AE-box, TCT-motif, Box 4, GATA-motif, G-box, GT1-motif, I-box, ACE, and TCCC-motif. Notably, the G-box element was present in every *TaCOL* gene and was the predominant element in the light-responsive category. In the phytohormone-responsive category, we identified cis-acting elements associated with specific hormone responses: abscisic acid (ABA) response (ABRE), auxin responsiveness (TGACG/CGTCA-motif), gibberellin (GA) response (TATC-box, P-box, and GARE-motif), methyl jasmonate (MeJA) responsiveness (TGACG/CGTCA-motif), and salicylic acid (SA) response (TCA-element). Among these, ABRE and TGACG/CGTCA-motif were the most frequent, with 255 and 128 occurrences, respectively. GA-responsive and SA-responsive elements were detected in 34 *TaCOL* members (49 total occurrences) and 16 *TaCOL* members (19 total occurrences), respectively. Stress-responsive elements were also abundant, including those associated with anaerobic conditions (ARE and GC-motif), drought stress (MYB, MYC, MBS), low-temperature stress (LTR), and defense/stress responses (TC-rich repeats). Seven *TaCOL* genes (*Ta-2B-COL4*, *Ta-6A-COL20*, *Ta-6B-COL26*, *Ta-6D-COL30*, *Ta-7D-COL44*, *Ta-7D-COL46*, and *Ta-U-COL51*) contained no fewer than 20 stress-responsive elements (**Figure 5B**). Drought-related MYB and MYC elements were particularly prominent: MYB elements occurred 358 times across 50 *TaCOLs*, while MYC elements occurred 174 times across 47 *TaCOLs*. Low-temperature-responsive LTR elements were identified 48 times in 29 *TaCOLs*. These findings suggested that *TaCOL* genes might play crucial regulatory roles in plant growth, development, and stress adaptation.

### 3.5 Spatiotemporal expression profile of *TaCOL* genes

**Figure 6:**
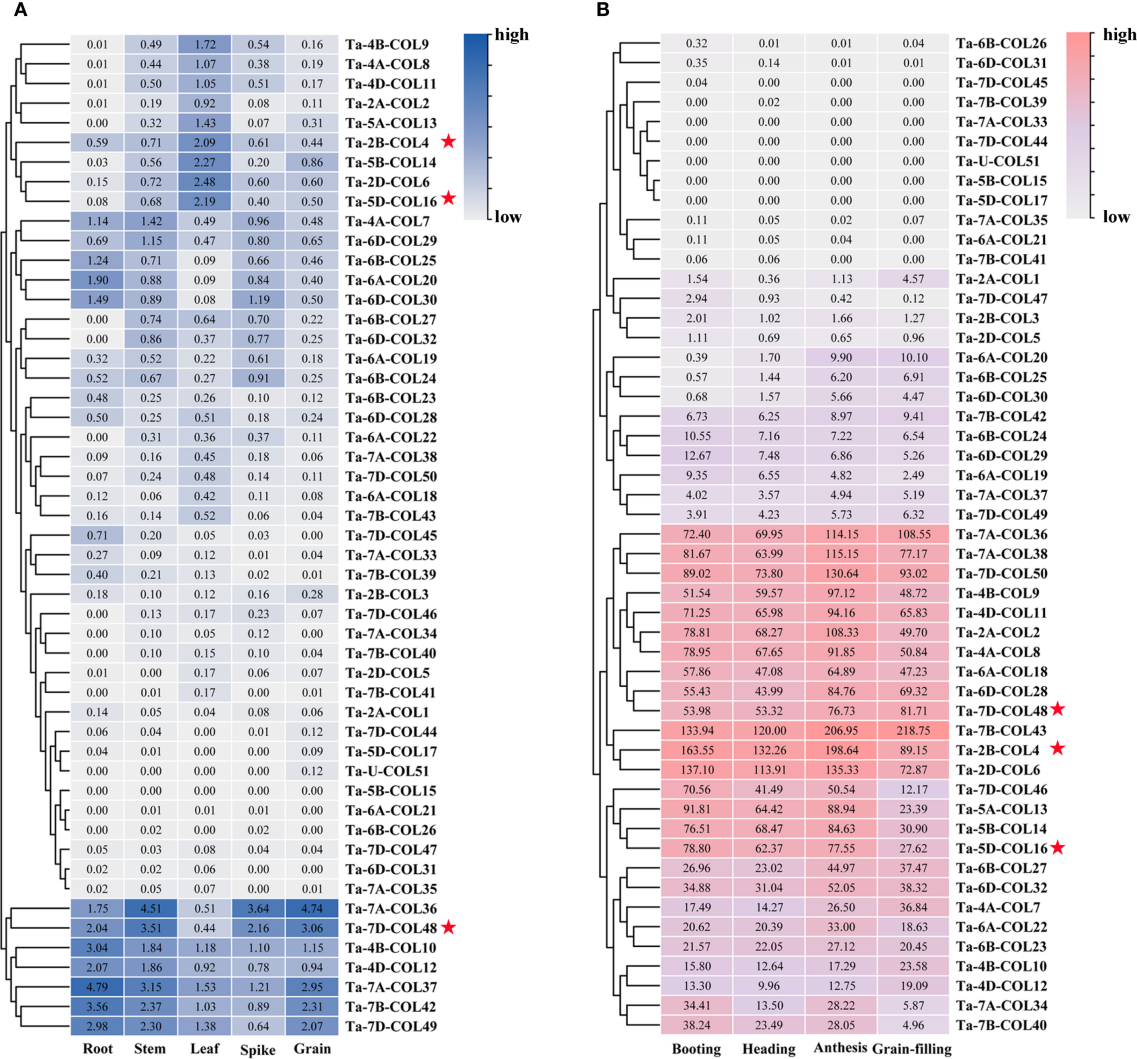
Expression analysis of TaCOL genes across five tissues and four developmental stages. **(A)** The expression levels of *TaCOL* genes were analyzed in five different tissues (root, stem, leaf, spike, and grain) and **(B)** across four developmental stages (booting, heading, anthesis, and grain-filling). Numerical values represented TPM values, and the legend indicated relatively high and low expression levels. The three *TaCOL* genes marked with red stars were selected for subsequent functional analysis.

To evaluate the expression patterns of *TaCOL* genes across five tissues (root, stem, leaf, spike, and grain) and four developmental stages (booting, heading, anthesis, and grain-filling), transcriptome data revealed that over half of the *TaCOL* genes showed no detectable expression levels (**Figure 6**), a common characteristic of transcription factors. Notably, seven *TaCOL* genes, such as *Ta-7A-COL36*, *Ta-7D-COL48*, *Ta-4B-COL10*, *Ta-4D-COL12*, *Ta-7A-COL37*, *Ta-7B-COL42*, and *Ta-7D-COL49.* showed high expression across all five tissues. However, some genes displayed tissue-specific expression. For instance, *Ta-4B-COL9*, *Ta-4A-COL8*, *Ta-4D-COL11*, *Ta-2A-COL2*, *Ta-5A-COL13*, *Ta-2B-COL4*, *Ta-5B-COL14*, *Ta-2D-COL6*, and *Ta-5D-COL16* had relatively higher expression in leaves than the other four tissues (**Figure 6A**). Additionally, 13 *TaCOL* genes, including *Ta-7A-COL36*, *Ta-7A-COL38*, *Ta-7D-COL50*, *Ta-4B-COL9*, *Ta-4D-COL11*, *Ta-2A-COL2*, *Ta-4A-COL8*, *Ta-6A-COL18*, *Ta-6D-COL28*, *Ta-7D-COL48*, *Ta-7B-COL43*, *Ta-2B-COL4*, and *Ta-2D-COL6*, showed particularly high expression levels across all four developmental stages. Notably, the expression level of *Ta-7B-COL43* exceeded 100. Furthermore, the expression levels of *Ta-7D-COL46*, *Ta-5A-COL13*, *Ta-5B-COL14*, and *Ta-5D-COL16* showed higher expression during the booting, heading, and anthesis stages than during the grain-filling stage (**Figure 6B**). Based on these findings, *Ta-2A-COL2*, *Ta-2B-COL4*, *Ta-2D-COL6*, *Ta-4A-COL8*, *Ta-4B-COL9*, *Ta-4D-COL11*, *Ta-5A-COL13*, *Ta-5D-COL16*, *Ta-7A-COL36*, and *Ta-7D-COL48* might play crucial roles in wheat growth and development. We also observed that *Ta-6A-COL20*, *Ta-6B-COL25*, and *Ta-6D-COL30* showed higher expression levels during anthesis and grain-filling stages, being three times higher than those during the booting and heading stages. This suggested that these genes might play important roles in the later stages of wheat reproductive development.

### 3.6 Transcriptome level of *TaCOLs* under abiotic stress

**Figure 7:**
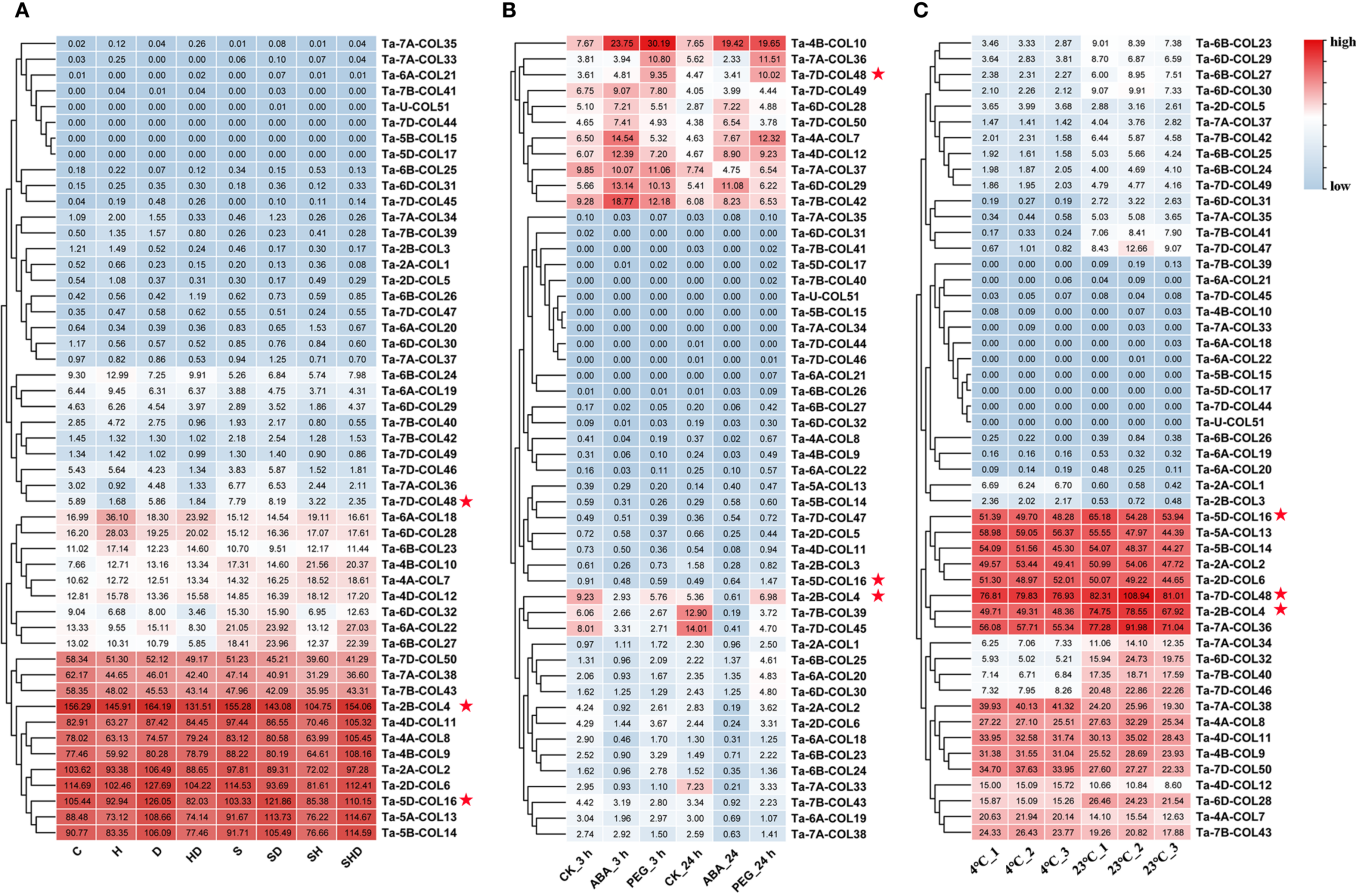
Heatmap of *TaCOLs* expression level under different abiotic stress treatments. **(A)** Expression levels of *TaCOL* genes in response to drought, heat, salt, and their combined stresses were presented in the heatmap. **(B)** Expression levels of *TaCOL* genes under PEG and ABA treatments at 3 h and 24 h were presented in the heatmap. **(C)** Expression levels of *TaCOL* genes at 4°C and 23°C were presented in the heatmap. The three *TaCOL* genes marked with red stars were selected for subsequent functional analysis.

To assess the expression patterns of *TaCOL* genes under diverse abiotic stress conditions, we conducted cluster analysis and visualized the results via heatmaps. The analysis revealed distinct and varied expression profiles across different treatments (**Figure 7A**). Notably, 12 *TaCOL* genes, namely *Ta-7D-COL50*, *Ta-7A-COL38*, *Ta-7B-COL43*, *Ta-2B-COL4*, *Ta-4D-COL11*, *Ta-4A-COL8*, *Ta-4B-COL9, Ta-2A-COL2*, *Ta-2D-COL6*, *Ta-5D-COL16*, *Ta-5A-COL13*, and *Ta-5B-COL14*, maintained sustained high transcription levels across all treatments, including the control. Furthermore, some *TaCOL* genes displayed coordinated transcriptional responses both individual and combined stress treatments. For example, *Ta-6A-COL18*, *Ta-6D-COL2*, *Ta-6B-COL23*, *Ta-4B-COL10*, *Ta-4A-COL7*, and *Ta-4D-COL12* were moderately upregulated under heat and drought stress compared to the control, with similar trends observed under combined stress. Other genes, such as *Ta-6D-COL32*, *Ta-6A-COL22*, and *Ta-6B-COL27*, showed comparable transcription patterns.

In an additional transcriptome analysis, we investigated the transcription levels of *TaCOL* genes under PEG-induced osmotic stress and ABA treatments (**Figure 7B**). Among these, *Ta-4B-COL10* was significantly induced, showing more than a threefold increase in expression following both ABA and PEG treatments at 3 h and 24 h. Conversely, *Ta-7B-COL39* and *Ta-7D-COL45* were markedly suppressed under the same conditions. *Ta-7A-COL36* and *Ta-7D-COL48* were upregulated in response to PEG but remained unresponsive to ABA. Additionally, *Ta-7D-COL49*, *Ta-6D-COL28*, and *Ta-7D-COL50* exhibited moderate expression levels that were unaffected by either ABA or PEG treatment.

**Figure 8:**
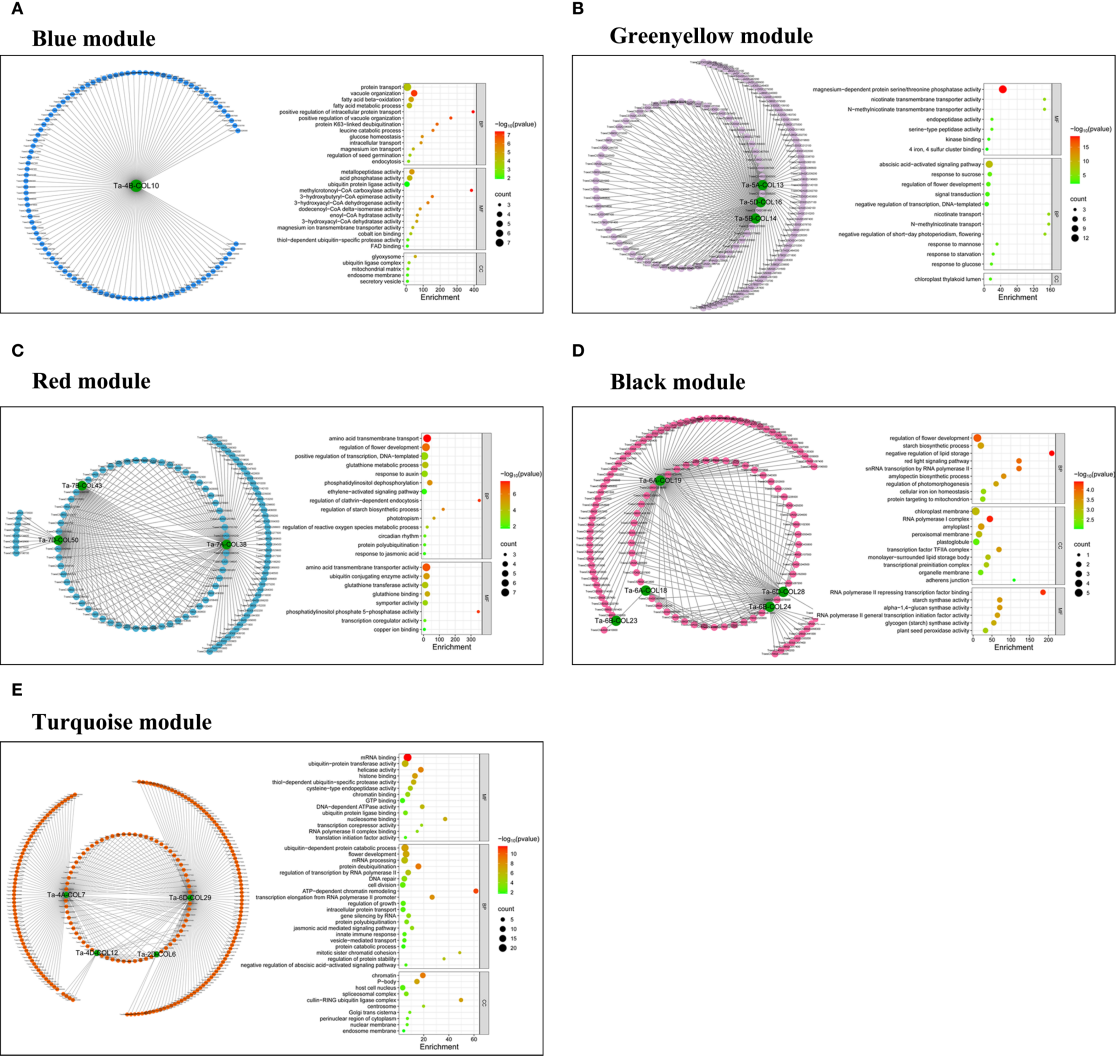
Co-expression networks and GO-enrichment analyses for different modules. **(A)** Co-expression network and GO-enrichment bubble plot for the top 100 genes (ranked by weight) in the blue module, using *Ta-4B-COL10* as the hub gene. In the bubble plot, the x-axis indicated the enrichment level as the ratio of gene proportions in the test set to the background set. The y-axis listed Gene Ontology annotations across BP, CC, and MF categories. **(B)** Co-expression network and GO-enrichment bubble plot for the top 200 genes in the greenyellow module, using *Ta-5A-COL13*, *Ta-5B-COL14*, and *Ta-5D-COL16* as the hub genes. The bubble plot format was the same as in **(A)**. **(C)** Co-expression network and GO-enrichment bubble plot for the top 200 genes in the red module, using *Ta-7A-COL38*, *Ta-7B-COL43*, and *Ta-7D-COL50* as the hub genes. The bubble plot followed the format in **(A)**. **(D)** Co-expression network and GO-enrichment bubble plot for the top 200 genes in the black module, using *Ta-6A-COL18*, *Ta-6A-COL19*, *Ta-6B-COL23*, *Ta-6B-COL24*, and *Ta-6D-COL28* as the hub genes. The bubble plot format was consistent with **(A)**. **(E)** Co-expression network and GO-enrichment bubble plot for the top 300 genes in the turquoise module, using *Ta-2D-COL6*, *Ta-4A-COL7*, *Ta-4D-COL12*, and *Ta-6D-COL29* as the hub genes. The bubble plot format matched that in **(A)**.

Temperature stress was also found to markedly influence the expression of *TaCOL* genes, consistent with its critical role in plant growth, development, and flowering time regulation. *Ta-2A-COL1* and *Ta-2B-COL3* were significantly induced under low-temperature conditions, while *Ta-4A-COL7*, *Ta-4D-COL12*, and *Ta-7B-COL43* showed moderate upregulation at 4°C. Interestingly, several *TaCOL* genes exhibited higher expression levels at 23°C, including *Ta-6D-COL32*, *Ta-7A-COL34*, *Ta-7B-COL40*, *Ta-7D-COL46*, *Ta-6B-COL23*, *Ta-6D-COL29*, *Ta-6B-COL27*, *Ta-6D-COL30*, *Ta-7A-COL37*, and *Ta-7B-COL42* (**Figure 8C**). These findings underscored the potential functional diversity of *TaCOL* genes in mediating plant developmental processes and adaptive responses to environmental stresses.

### 3.7 Co-expression network analysis

To dissect the transcriptional regulation of *TaCOLs* under individual and combinatorial drought, heat, and salt stresses, we constructed a weighted gene co-expression network from high-quality transcriptome data. After stringent filtering (mean TPM ≥ 1 and row sum ≥ 28), 35574 genes were retained. A scale-free topology was achieved with a soft-thresholding power of β = 11 (**Supplementary Figure S2B**). Weighted Gene Co-expression Network Analysis (WGCNA) resolved 10 modules of highly co-expressed genes (**Supplementary Figure S2D**), among which 25 *TaCOLs* were distributed across five distinct modules. To visualize the co-expression network with *TaCOLs* as central nodes, we extracted the top 100 (blue), 200 (black, red, green-yellow), and 300 (turquoise) genes exhibiting the highest topological overlap matrix values from each module, retaining a final set of 16 *TaCOL* genes. The blue module, which contains *Ta-4B-COL10*, was enriched for protein transport and fatty-acid metabolism (**Figure 8A**). Three *TaCOLs* (*Ta-5A-COL13*, *Ta-5B-COL14*, and *Ta-5D-COL16*), in the greenyellow module were associated with Mg^2+^-dependent serine/threonine phosphatase activity and ABA signaling (**Figure 8B**). The red module, harboring *Ta-7A-COL38*, *Ta-7B-COL43*, and *Ta-7D-COL50*, was linked to amino-acid transmembrane transport and floral development (**Figure 8C**). In the black module, five *TaCOLs* (*Ta-6A-COL18/19*, *Ta-6B-COL23/24*, *Ta-6D-COL28*) converged on flower development, starch biosynthesis, and chloroplast-membrane organization (**Figure 8D**). The turquoise module, comprising *Ta-2D-COL6*, *Ta-4A-COL7*, *Ta-4D-COL12*, and *Ta-6D-COL29*, was enriched for ubiquitin-dependent protein catabolism, floral development, and mRNA processing (**Figure 8E**). KEGG pathway annotations for each module were provided in **Supplementary Figure S3**.

### 3.8 Expression levels analysis of *TaCOLs* under long and short photoperiod

**Figure 9:**
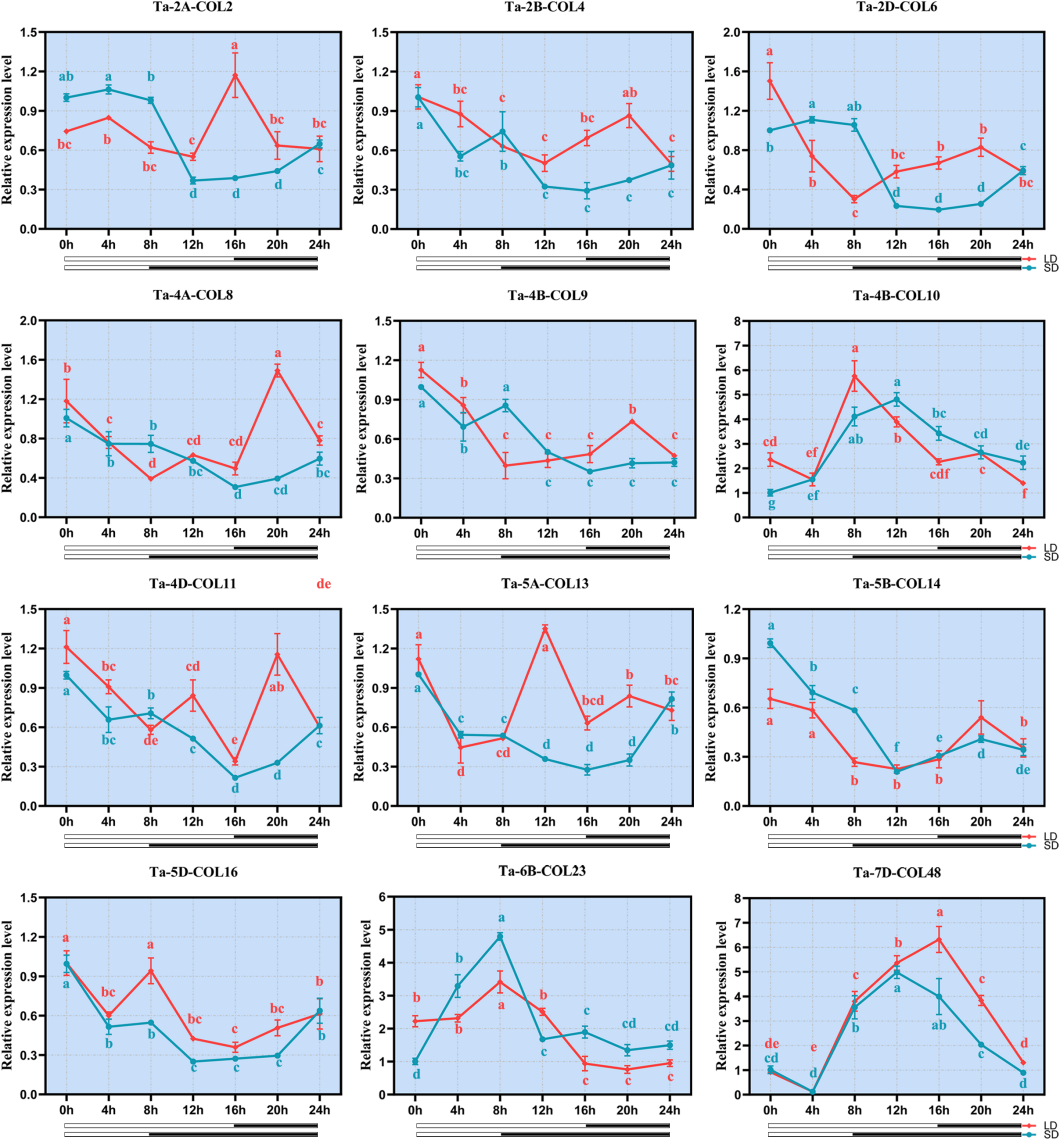
qRT-PCR expression analysis of 12 *TaCOLs* under long-day and short-day treatments. The y-axis represented the relative expression levels. The x-axis indicated the time course of light-stress treatments for each *TaCOL* gene. Bars indicated the standard deviations (SD) from three biological replicates.

Based on the consistently high expression profiles in available transcriptome data across multiple tissues, developmental stages, and stress treatments; while also ensuring representation from all three phylogenetic subfamilies, we monitored the expression profiles of 12 *TaCOL* genes under short-day (SD) and long-day (LD) conditions to explore their potential roles in photoperiodic regulation. Notably, *Ta-4B-COL10*, *Ta-6B-COL23* and *Ta-7D-COL48* were sharply up-regulated under both photoperiods, whereas the remaining nine genes were globally repressed by light (**Figure 9**). Among the latter, *Ta-2B-COL4*, *Ta-2D-COL6*, *Ta-4B-COL9*, *Ta-4D-COL11*, *Ta-5B-COL14* and *Ta-5D-COL16* maintained low, albeit fluctuating, transcript levels throughout the time-course. *Ta-2A-COL2*, *Ta-4A-COL8* and *Ta-5A-COL13* declined progressively under SD, but exhibited discrete peaks under LD. Interestingly, seven *TaCOLs* (*Ta-2B-COL4*, *Ta-4B-COL9*, *Ta-4B-COL10*, *Ta-5B-COL14*, *Ta-5D-COL16*, *Ta-6B-COL23* and *Ta-7D-COL4*8) displayed near-identical expression trajectories in SD and LD, indicating a photoperiod-independent mode of regulation.

### 3.9 Subcellular localization and transactivation activity of three TaCOLs

**Figure 10:**
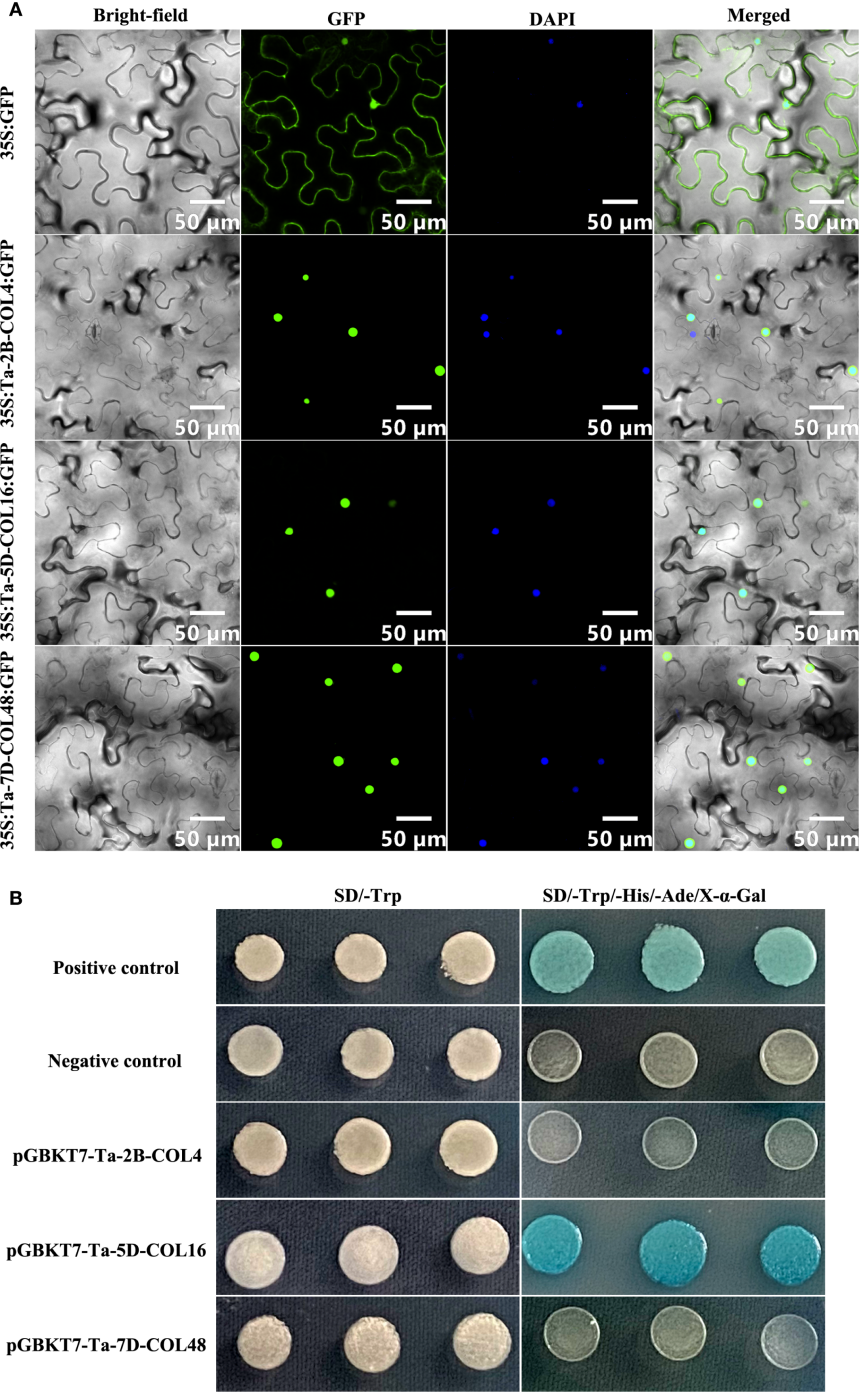
The subcellular localization and yeast transcriptional activity assay of Ta-2B-COL4, Ta-5D-COL16, and Ta-7D-COL48. **(A)** The image showed the location of GFP and TaCOL-GFP proteins in bright-field, fluorescence channel, DAPI channel, and the merged diagram. Scale bar = 50 μm. **(B)** The growth assay of Ta-2B-COL4, Ta-5D-COL16, and Ta-7D-COL48 proteins on SD/-Trp medium and SD/-Ade/-His/-Trp/X-a-gal medium.

Expression analysis under temperatures of 4°C and 23°C, as well as across five tissues and four developmental stages, revealed that *Ta-2B-COL4*, *Ta-5D-COL16*, and *Ta-7D-COL48* exhibited stable and high expression levels (**Figures 6**, **7**; **Supplementary Table S6**). Based on these findings, these three genes were selected for sub-cellular-localization and trans-activation assays. The coding sequences without stop codons, were fused to GFP constructs. These constructs were expressed in *Nicotiana benthamiana* leaves alongside a 35S promoter-driven GFP control. Whereas 35S::GFP fluorescence was distributed throughout the cell, the three CONSTANS-like proteins were distinctly localized in the nucleus (**Figure 10A**), in agreement with the predictions provided on the website (**Supplementary Table S1**). In addition, to evaluate transcriptional activity, each pGBKT7-TaCOL construct was individually introduced into Y2HGold yeast, together with the positive control (pGBKT7-53 + pGADT7-T) and the negative control (pGBKT7). All transformants produced white colonies on SD/-Trp medium. On SD/-Ade/-His/-Trp/X-a-gal medium, only yeast cells with Ta-5D-COL16 grew well and turned blue, similar to the positive control, whereas Ta-2B-COL4 and Ta-7D-COL48 failed to grow on this medium, indistinguishable from the negative control (**Figure 10B**). Thus, Ta-5D-COL16 activated both the HIS3 and LacZ reporters, demonstrating its self-transcriptional activity in yeast.

## 4 Discussion

The *CONSTANS-like* (*COL*) gene family has been characterized in numerous species, yet it remains understudied in wheat. Here, we identified 51 *CONSTANS-like* members in the Chinese Spring reference cultivar. To trace their evolutionary origin, we compiled 116 CONSTANS-like proteins from *A. thaliana* (17), *O. sativa* (16), *Z. mays* (18) and *P. edulis* (14) and *T. aestivum* (51) and classified them into three subgroups following the criteria established by Hu et al. (2018) (**Supplementary Figure S1**). Consequently, the 51 wheat CONSTANS-like proteins were assigned to Subfamilies I, II, and III based on the single phylogenetic tree presented in **Figure 2A**. Nevertheless, our tripartite classification deviated slightly from the canonical B-box criterion proposed by Griffiths et al. (2003): subfamily I TaCOLs possess two B-box motifs, a CCT domain and the VP motif required for COP1 interaction; subfamily II members contain a single B-box together with a CCT domain; whereas subfamily III TaCOLs harbor a complete B-box that is distinct from those of subfamilies I and II, along with the CCT domain (Khatun et al., 2021). All TaCOL proteins in this study harbored motif 1 (the CCT domain) plus motif 2 and/or motif 3, both of which corresponded to the B-box domain (**Figure 2A**). Nevertheless, the number of B-box repeats was not strictly subfamily-specific. Among the 18 subfamily I members, Ta-6D-COL28, Ta-6A-COL18 and Ta-6B-COL23 possessed only one B-box, whereas the remainder contained two. Conversely, five subfamily II members unexpectedly carried two B-box repeats; this deviation coincided with the acquisition of a unique, conserved motif 4 (**Figure 2A**; 2B). Subfamily III proteins uniformly contained a single B-box and a CCT module, yet sequence divergence within this group was comparable to that observed between subfamilies I and II (**Figure 2B**; **Supplementary Table S2**). Exon-intron architecture further underscored the heterogeneity within each subfamily (**Figure 2C**). Subfamily-II genes showed typically four-exon structures, but *Ta-4B-COL10* (three exons) and *Ta-4A-COL7* (five exons) had notable exceptions. Phylogenetic analysis indicated that the two genes were homoeologues (**Figure 3A**; **Supplementary Table S3**). It was speculated that *Ta-4B-COL10* might have lost an exon due to splicing site mutations or exon skipping, while *Ta-4A-COL7* might have increased regulatory complexity by acquiring additional exons through intron-exon insertion or transposon element insertion (Wang et al., 2021). Meanwhile, *Ta-4A-COL7* showed high expression at 23°C but a rapid decrease at 4°C, whereas *Ta-4B-COL10* exhibited no significant expression at either temperature (**Figure 7C**).

In present study, a total of 51 *TaCOL* genes were identified in Chinese Spring wheat, a significantly higher number than that found in other species, such as *H. vulgare* (9), *O. sativa* (16) (Griffiths et al., 2003), *S. italica* (11) (Jiang et al., 2024), *Z. mays* (19) (Song et al., 2018), *A. thaliana* (17) (Khanna et al., 2009), *Cucumis sativus* (12) (Tian et al., 2021), *Solanum lycopersicum* (13) (Yang et al., 2020), and *Solanum tuberosum* (15) (Li et al., 2023). Notably, the number of *TaCOL* genes in wheat was three times higher than that in *A. thaliana*. This significant difference might be attributed to the two rounds of whole-genome duplication events in hexaploid wheat (Cavalet-Giorsa et al., 2024). As a result of polyploidization, copies of each *CONSTANS-like* gene were preserved across the A, B, and D subgenomes, establishing a triplicate genomic foundation. In terms of chromosomal distribution, both the A and B genomes contained 16 *TaCOL* genes, while the D genome had 18 *TaCOL* genes (**Figure 4A**). Overall, the *TaCOL* gene family has remained largely conserved throughout the reorganization and evolution of the wheat genome. This conservation may be attributed to the retention of extra gene copies of *TaCOL* through duplication events, which likely enhances wheat’s environmental adaptability (Huo et al., 2018).

In allohexaploid wheat, the expansion of gene families stems from the combined effects of whole-genome duplication (WGD), localized duplications, transposon activity, and natural selection (He et al., 2022; Thomas et al., 2014). To investigate the expansion of the *COL* gene family in wheat, we detected 64 segmental duplication pairs among the 51 *TaCOL* genes. This suggested that segmental duplication played a pivotal role in the expansion of this gene family during evolution, a pattern also observed in duplication models of *foxtail millet* (Jiang et al., 2024), potato (Li et al., 2023) and sunflower (Tianzeng et al., 2021). However, analysis of the *COL* genes in Chinese white pear revealed that tandem and proximal duplications occurred subsequent to WGD events (Cai et al., 2025). This might be attributed to the distinct evolutionary trajectories and rates across different species (Qiao et al., 2019). Thus, the *COL* gene family has undergone a higher frequency of gene duplication events throughout its evolutionary history, as indicated by the Ks values and Ka/Ks ratios (**Figure 3B**; **Supplementary Table S3**). Meanwhile, we further explored the evolutionary distance of *COL* genes between hexaploid wheat and eight other Poaceae species through interspecies collinearity analysis. The mean Ks values, ordered from smallest to largest, were as follows: Td-Ta (0.203016013), Ae-Ta (0.26228061), Hv-Ta (0.281602287), Sc-Ta (0.293103976), Si-Ta (0.527560353), Os-Ta (0.595051996), Sb-Ta (0.602337798), and Zm-Ta (0.652450481). The ascending Ks series precisely mirrors the stepwise phylogenetic distance between hexaploid wheat and the eight grasses (**Figure 4D**). *T. dicoccoides*, *A. tauschii*, *S. cereale*, and *H. vulgare* are the closest relatives to hexaploid wheat, while *S. italica*, *S. bicolor*, and *Z. mays* are more distant. Moreover, the analysis of species divergence timing reveals that hexaploid wheat (AABBDD), which originated from the hybridization of *T. dicoccoides* (AABB) and *A. tauschii* (DD) approximately 8000 years ago (Huang et al., 2002; Wang et al., 2013), diverged from *S. cereale* around 3–4 million years ago (MYA) (Zhao et al., 2023), from barley around 8–9 MYA (Liu et al., 2020b), and from rice around 49.3-58.9 MYA (Wang et al., 2023). Additionally, Panicoideae subfamily species such as *S. bicolor*, *S. italica*, and *Z. mays* diverged from the wheat group approximately 57–60 MYA (Zhao et al., 2023). Thus, pairwise Ks distributions not only recapitulate the known species tree but also provide an independent, molecular clock-based timeline for the evolution of the *COL* family in the Triticeae and beyond.

To gain deeper insights into the functions of *TaCOLs*, we utilized transcriptomic datasets derived from drought, heat, salt stress conditions, and their combinations to perform co-expression network analysis. This approach enabled us to investigate two key aspects: first, identifying which *TaCOL* genes function within the same regulatory module; and second, predicting the potential functions of genes across distinct modules (Zhang et al., 2022a; Yang et al., 2025). Strikingly, our analysis revealed that only 16 *TaCOLs* were distributed across five regulatory modules (**Figure 8**). The turquoise, black, and red modules were enriched in flower development and flowering-related genes, suggesting that *TaCOLs* transmit stress signals to accelerate flowering, a classic drought-escape strategy observed in long-day cereals (Mahrookashani et al., 2017) Within the greenyellow module, three *TaCOL* genes co-express with ABA-responsive element binding factors (ABFs) and NCED (9-cis-epoxycarotenoid dioxygenase), supporting a model in which ABA accumulation under water deficit feeds forward on *TaCOL* expression to fine-tune stomatal closure and developmental timing (**Figure 7B**; Zareen et al., 2024; Zhang et al., 2020). Indeed, the expression patterns of *TaCOLs* under PEG-induced osmotic stress and ABA treatment were found to be synergistic (**Figure 7B**). Consequently, we propose that in unfavorable environmental conditions, stress responses are mediated through the ABA pathway, which modulates wheat growth and developmental processes to preserve its genetic heritability.

Wheat is a typical long-day plant. Based on our previous survey of high-expression *TaCOL* candidates from tissue-specific and abiotic stress transcriptomes, we selected 12 *TaCOLs* for qRT-PCR analysis to assess their expression levels under long and short photoperiods. The results showed that *Ta-4B-COL10*, *Ta-6B-COL23*, and *Ta-7D-COL48* were significantly induced by light and exhibited similar expression patterns under both short and long photoperiods (**Figure 9**), indicating they were light-responsive but not photoperiod-sensitive. This characteristic was analogous to that of *AtCOL3* and *OsCO3* which integrated UV-A/blue light signals via CRY1 (cryptochrome 1) and mediate rapid FT (FLOWERING LOCUS T) induction under inductive photoperiods (Kim et al., 2008; Chen et al., 2021). Whether wheat orthologues of these *COL* genes act through the same CRY-COL-FT signaling cascade awaits verification via further experiments. It should be noted that the current expression data are derived from a 24-hour time course. While this provides initial insights into diurnal expression patterns, further validation across multiple consecutive cycles would be valuable to confirm the robustness and rhythmic characteristics of their light responsiveness.

## 5 Conclusion

In this study, we identified 51 *CONSTANS-like* genes in Chinese Spring wheat and systematically classified them into three distinct subfamilies. Comprehensive phylogenetic analysis, along with examination of gene structure and motif composition, significantly improved our understanding of the organizational and evolutionary features of the *TaCOL* gene family. Further investigation into gene duplication events and collinearity relationships provided critical insights into the expansion mechanisms and evolutionary trajectory of these genes. Additional promoter analyses, expression profiling, and co-expression network studies offered functional clues, suggesting roles in stress adaptation and flowering regulation. Notably, we demonstrated that Ta-2B-COL4, Ta-5D-COL16, and Ta-7D-COL48 were nuclear-localized and likely served pivotal roles in stress responses and the regulation of flowering. Overall, these findings provide a robust foundation for elucidating the functional evolution of *COL* genes in wheat, and offer valuable genetic resources for future research aimed at enhancing wheat adaptability and resilience in the face of environmental challenges.

## Data Availability

The original contributions presented in the study are included in the article/**Supplementary Material**. Further inquiries can be directed to the corresponding author/s.

## Supplementary material

Supplementary Figure 1 A comprehensive phylogenetic tree of 116 CONSTANS-like protein sequences from *A. thaliana* (17), *O. sativa* (16), *Z. mays* (18) and *P. edulis* (14) and *T. aestivum* (51). The phylogenetic analysis of 116 proteins was performed using MEGA11 software using neighbor-joining method with a bootstrap analysis of 1000 replicates.

Supplementary Figure 2 Diagrams associated with the construction of co-expression networks. A. Sample clustering dendrograms: the original sample dendrogram (grouped by similarity to identify outliers) and the dendrogram after outlier removal (refined sample clustering). B. “Scale independence” plot: the x-axis represented soft-thresholding power, the y-axis corresponded to the scale-free fitting index, and the red line indicated the criterion for soft threshold selection. C. Cluster dendrogram displaying gene/sample clustering: “Height” indicated the merge distance, and “Module colors” designated groups with similar expression patterns. D. Eigengene adjacency heatmap: a color gradient from blue to red reflected the correlation levels among module eigengenes, ranging from low to high.

Supplementary Figure 3 Bar charts of KEGG enrichment analysis for five modules. The genes involved in each module were consistent with those in **Figure 8**.

Supplementary Table 1 The detailed information about 51 predicted *TaCOLs* in *T. aestivum.*

Supplementary Table 2 Analysis and distribution of 10 conserved motifs among TaCOLs.

Supplementary Table 3 The Ka, Ks and Ka/Ks value of the paralogous and orthologous pairs of *TaCOLs.*

Supplementary Table 4 The cis-elements of *TaCOLs* in promoter regions contained plant growth/development, phytohormone response, and stress response-related.

Supplementary Table 5 The SRR number corresponding to wheat treatments.

Supplementary Table 6 Expression levels under different temperatures, across five tissues and four developmental stages.

Supplementary Table 7 The genes involved in the five modules and GO enrichment.

Supplementary Table 8 The primer sequence of qRT-PRC for 12 *TaCOLs.*

Supplementary Table 9 The primer sequence of vector construction for three *TaCOLs.*
