# Supplementary figures and images for: Genome-wide identification and characterization analysis of CONSTANS-like gene family in wheat (Triticum aestivum L.)
Source: Front Plant Sci. 2025 Oct 16;16:1646979. doi: 10.3389/fpls.2025.1646979 (PMC12573777; doi:10.3389/fpls.2025.1646979)

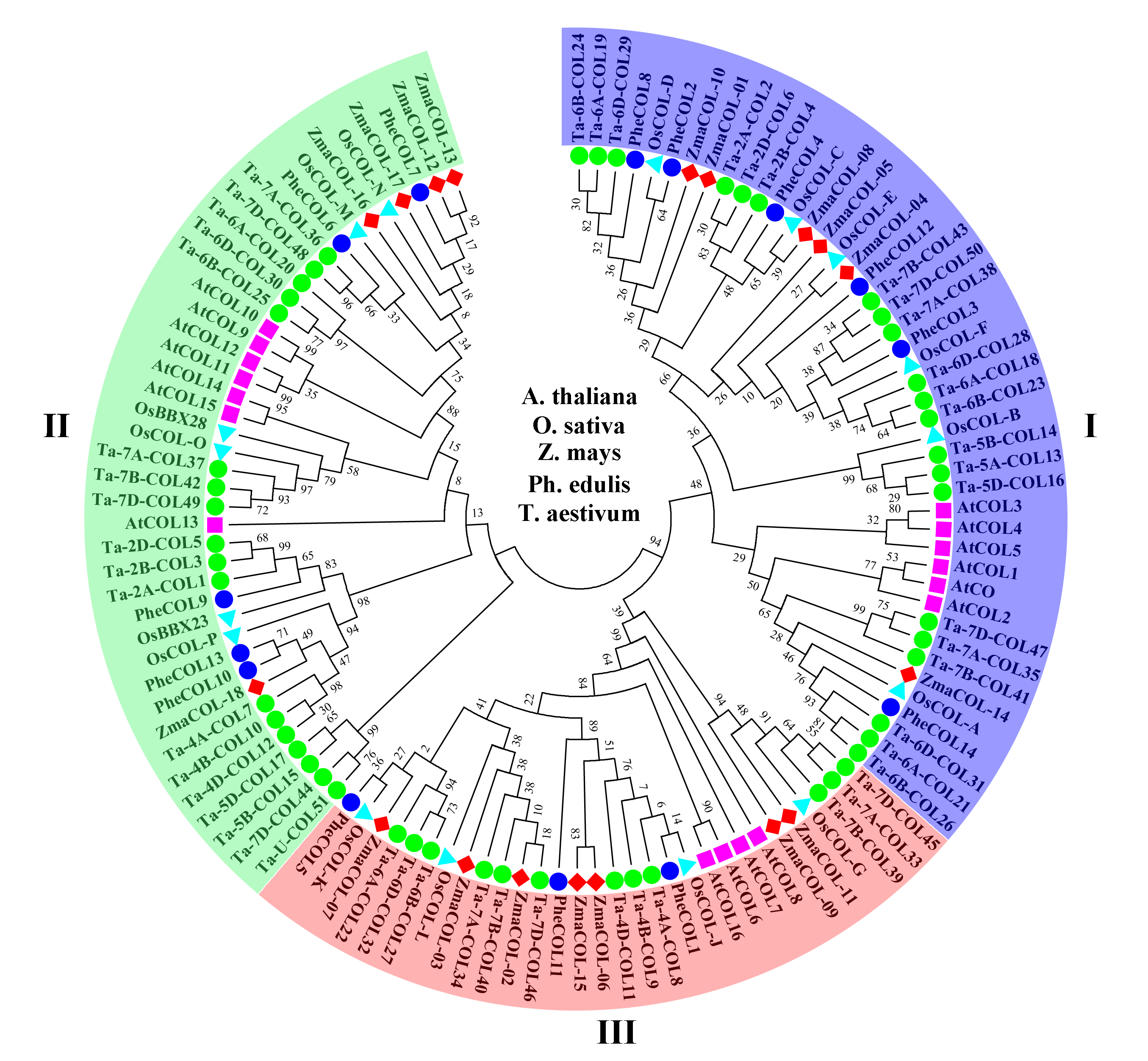

Supplement: Supplementary Figure 1 — A comprehensive phylogenetic tree of 116 CONSTANS-like protein sequences from A. thaliana (17), O. sativa (16), Z. mays (18) and P. edulis (14) and T. aestivum (51). The phylogenetic analysis of 116 proteins was performed using MEGA11 software using neighbor-joining method with a bootstrap analysis of 1000 replicates. [file Image1.tif]

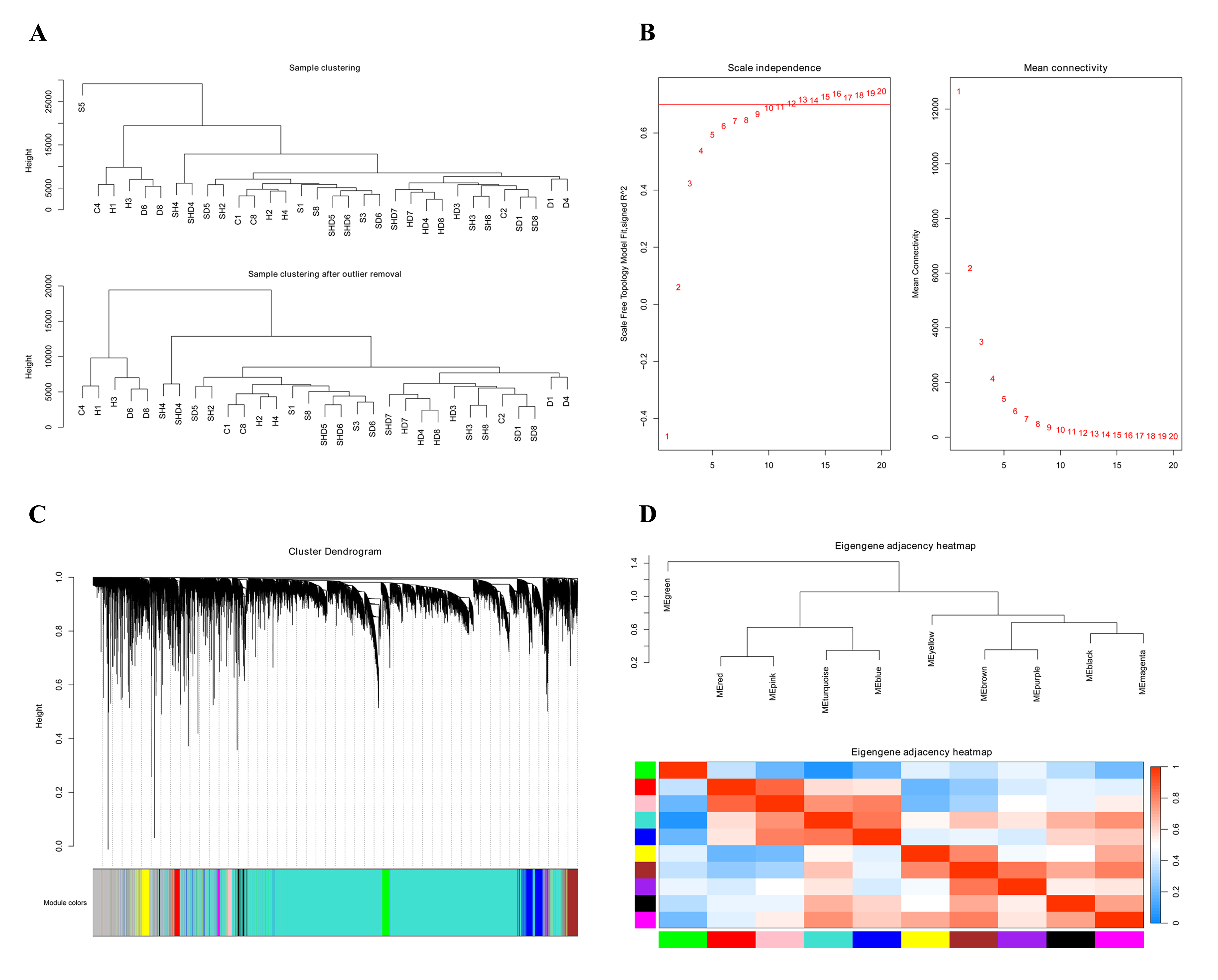

Supplement: Supplementary Figure 2 — Diagrams associated with the construction of co-expression networks. A. Sample clustering dendrograms: the original sample dendrogram (grouped by similarity to identify outliers) and the dendrogram after outlier removal (refined sample clustering). B. “Scale independence” plot: the x-axis represented soft-thresholding power, the y-axis corresponded to the scale-free fitting index, and the red line indicated the criterion for soft threshold selection. C. Cluster dendrogram displaying gene/sample clustering: “Height” indicated the merge distance, and “Module colors” designated groups with similar expression patterns. D. Eigengene adjacency heatmap: a color gradient from blue to red reflected the correlation levels among module eigengenes, ranging from low to high. [file Image2.tif]

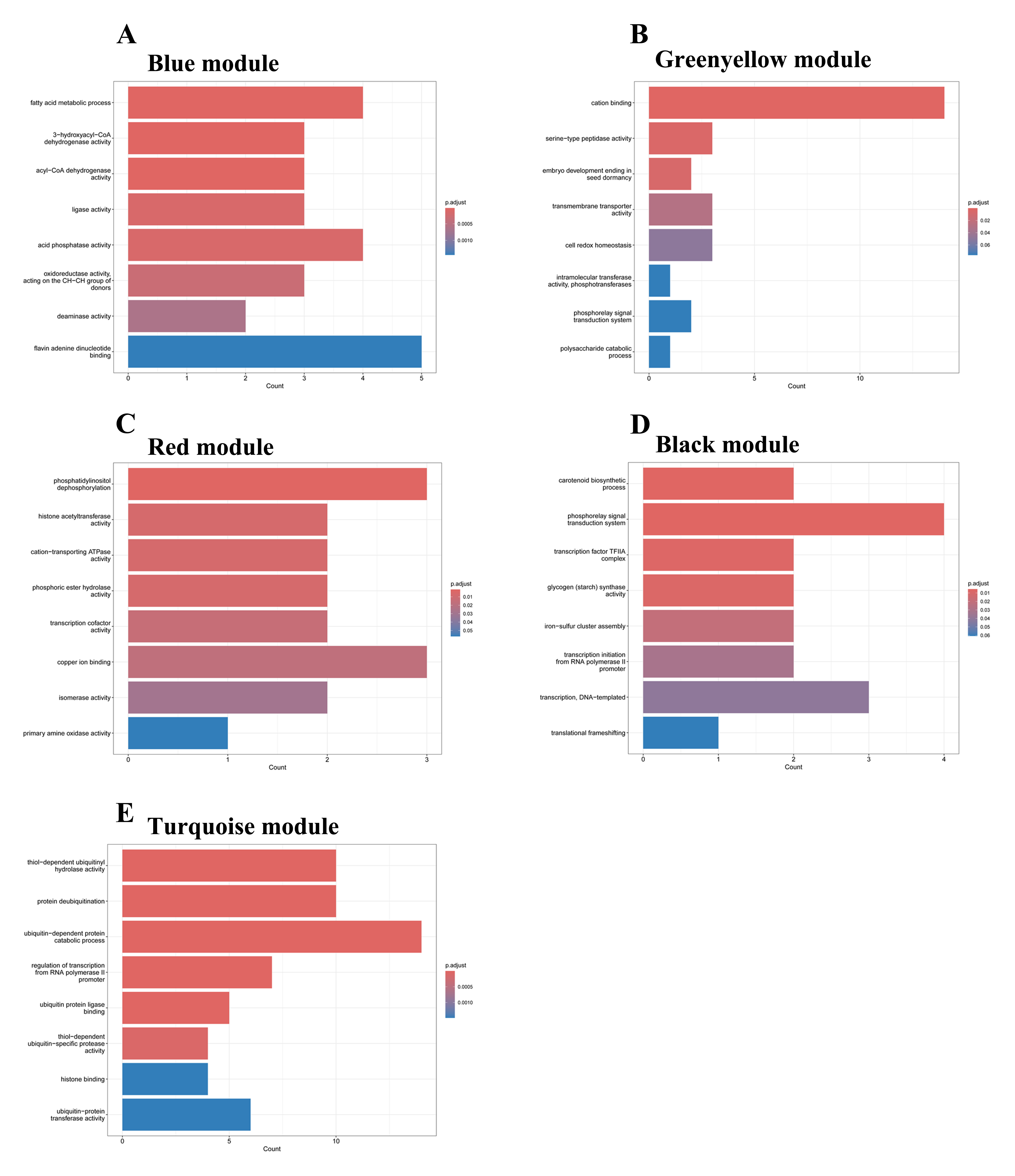

Supplement: Supplementary Figure 3 — Bar charts of KEGG enrichment analysis for five modules. The genes involved in each module were consistent with those in Figure 8 . [file Image3.tif]
